# Flagella-Driven Motility of Bacteria

**DOI:** 10.3390/biom9070279

**Published:** 2019-07-14

**Authors:** Shuichi Nakamura, Tohru Minamino

**Affiliations:** 1 Department of Applied Physics, Graduate School of Engineering, Tohoku University, 6-6-05 Aoba, Aoba-ku, Sendai 980-8579, Japan; 2 Graduate School of Frontier Biosciences, Osaka University, 1-3 Yamadaoka, Suita, Osaka 565-0871, Japan

**Keywords:** bacterial flagellum, chemotaxis, ion motive force, ion channel, mechanochemical coupling, molecular motor, motility, torque generation

## Abstract

The bacterial flagellum is a helical filamentous organelle responsible for motility. In bacterial species possessing flagella at the cell exterior, the long helical flagellar filament acts as a molecular screw to generate thrust. Meanwhile, the flagella of spirochetes reside within the periplasmic space and not only act as a cytoskeleton to determine the helicity of the cell body, but also rotate or undulate the helical cell body for propulsion. Despite structural diversity of the flagella among bacterial species, flagellated bacteria share a common rotary nanomachine, namely the flagellar motor, which is located at the base of the filament. The flagellar motor is composed of a rotor ring complex and multiple transmembrane stator units and converts the ion flux through an ion channel of each stator unit into the mechanical work required for motor rotation. Intracellular chemotactic signaling pathways regulate the direction of flagella-driven motility in response to changes in the environments, allowing bacteria to migrate towards more desirable environments for their survival. Recent experimental and theoretical studies have been deepening our understanding of the molecular mechanisms of the flagellar motor. In this review article, we describe the current understanding of the structure and dynamics of the bacterial flagellum.

## 1. Introduction

Bacterial motility is an extremely intriguing topic from various scientific aspects. For example, motility can be a crucial virulence attribute for pathogenic bacteria, such as *Salmonella enterica* (hereafter referred to *Salmonella*) and *Helicobacter pylori* [1,2]. Bacterial motility also plays a significant role in mutualistic symbioses [3,4]. Furthermore, motile bacteria are also a representative example for understanding the underlying physical principles that form the basis of energy conversion, force generation and mechanochemical coupling mechanisms [5]. Active motilities of bacteria are represented by movement in liquid (e.g., swimming motility in *Escherichia coli* and *Salmonella*) and on solid surfaces (e.g., flagella-driven swarming motility in *Proteus mirabilis* and *Vibrio parahaemolyticus*, gliding motility in *Mycoplasma mobile*, and twitching motility in *Pseudomonas aeruginosa*), and passive motility is typically actin-based locomotion (e.g., *Listeria monocytogenes* and *Shigella* spp.) [6]. Since bacterial motility varies among bacterial species, bacteria utilize their own motility system optimized for their habitats.

**Figure 1:**
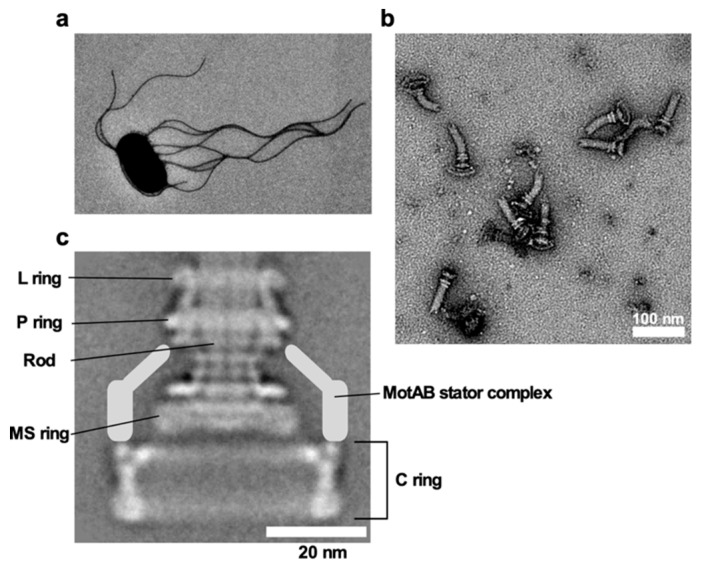
*Salmonella* flagellum. (**a**) Electron micrograph of *Salmonella* cell. The micrograph was taken at a magnification of ×1200. (**b**) Electron micrograph of hook-basal bodies isolated from *Salmonella* cells. (**c**) CryoEM image of purified basal body. Purified basal body consists of the L, P, MS and C rings and the rod. A dozen MotAB complex are associated with the basal body to act as a stator unit in the motor but is gone during purification.

*E. coli* and *Salmonella* use flagella viewable from the cell exterior as a thin, long, helical filament (Figure 1a). On the other hand, the flagella of spirochetes reside within the periplasmic space, and so they are called periplasmic flagella [7]. Whether the bacterial flagella are exposed to the cell exterior or are hidden within the cell body, the flagellum is divided into three structural parts: the basal body as a rotary motor, the hook as a universal joint and the filament as a molecular screw in common (Figure 1b), and flagellar formation and function involves more than 60 genes [8,9,10].

The bacterial flagellar motor is powered by the transmembrane electrochemical gradient of ions, namely ion motive force (IMF) and rotates the flagellar filament to generate thrust to propel the cell body. The maximum motor speed reaches 300 revolutions per second in *E. coli* and *Salmonella* [11] and 1700 revolutions per second in a marine bacterium *Vibrio alginolyticus* [12]. Thus, the rotational speed of the flagellar motor is much faster than that of a manufactured car engine such as formula one car. The flagellar motor is composed of a rotor and multiple stator units. Each stator unit acts as a transmembrane ion channel to conduct cations such as protons (H^+^) or sodium ions (Na^+^) and applies force on the rotor [13,14].

The flagellar motors of *E. coli* and *Salmonella* rotate in both counterclockwise (CCW) and clockwise (CW) without changing the direction of ion flow. *E. coli* and *Salmonella* cells can swim in a straight line by bundling left-handed helical filaments behind the cell body (run) when all of them rotate in CCW direction. When one or multiple motors switch the direction of rotation from CCW to CW, the flagellar bundle is disrupted, enabling the cell to tumble and change the swimming direction. *E. coli* and *Salmonella* cells sense temporal changes in nutrients, environmental stimuli, and signaling molecules to coordinate the switching frequency of the motor. Transmembrane chemoreceptors, energy-related taxis sensors and intracellular phosphotransferase systems detect environmental signals and then convert them into intracellular signals. Then, an intracellular signal transduction system transmits the signals to the flagellar motor to switch the direction of motor rotation from CCW to CW. The cells repeat a run–tumble pattern to explore more favorable environments for their survival [15]. This review article covers our current understating of flagella-driven motility mechanism in *E. coli* and *Salmonella*. We also describe the structural and functional diversities of the bacterial flagella.

## 2. Axial Structure

The axial structure of the bacterial flagellum is commonly a helical assembly composed of 11 protofilaments and is divided into at least three structural parts: the rod, the hook and the filament from the proximal to the distal end. The rod is straight and rigid against bending and twisting and acts as a drive shaft. The hook is supercoiled and flexible against bending and acts as a universal joint to smoothly transmit torque produced by the motor to the filament. The filament is also supercoiled but stiff against bending. The filament is normally a left-handed supercoil to act as a helical screw to produce thrust for swimming motility. The filament undergoes polymorphic transformation from the left-handed supercoil to right-handed ones when bacterial cells tumble and change swimming direction [16].

### 2.1. Flagella Filament

The flagellar filament of *E. coli* is formed by ~30,000 copies of flagellin, FliC. *Salmonella* has the *fljB* gene encoding another flagellin subunit in addition to the *fliC* gene. Because flagellin is a major target of host immune system (H-antigen), such an additional flagellin subunit enables *Salmonella* cells to escape from adaptive immune response of the host more efficiently compared to *E. coli* cells [17]. The FliC-type filament structure derived from *Salmonella* has been solved at the atomic level [18,19,20]. *Salmonella* FliC is composed of four domains D0, D1, D2 and D3, arranged from the inner to the outer part of the filament structure. Domains D0 and D1 are well conserved among bacterial species whereas domains D2 and D3 are variable even among *Salmonella* spp., because these two domains are the major targets of antibodies [21].The supercoiled forms of the filament structure are generated by combinations of two distinct left-handed (L-type) and right-handed (R-type) helical conformations of flagellin molecule and packing interactions of the L- and R-type protofilaments, and so the helical properties of each supercoil are determined by a ratio of L-type protofilaments to R-type ones in the filament structure [22,23]. The intermolecular distance along the L-type straight filament consisting of all L-type protofilaments is 0.8 Å longer than that of the R-type one composed of all R-type protofilaments [24]. Since a conformational change of a β-hairpin in domain D1 generates the 0.8 Å difference in repeat distance, this β-hairpin is thought to be responsible for the supercoiling switching [17]. Therefore, it seems likely that an abrupt reversal of motor rotation applies mechanical stress on each protofilament to induce the sliding motion between flagellin subunits along the protofilament, thereby changing the filament structure from the L-type supercoil to R-type one to disrupt the flagellar bundle for tumbling of the cell body [18]. Recent high-resolution electron cryomicroscopy (cryoEM) imaging analyses of L- and R-type straight filaments derived from *Bacillus subtilis* and *P. aeruginosa* have shown that the switching of the supercoiled forms of these flagellar filaments occurs in a way similar to the *Salmonella* filament [25].

Although the flagellar filaments of *E. coli* and *Salmonella* are formed by a single flagellin subunit, many bacterial species have multiple flagellins for the synthesis of flagellar filaments. The single polar flagellum of *Caulobacter crescentus* is composed of six flagellins, FljJ, FljK, FljL, FljM, FljN, and FljO [26]. Although the function of each flagellin subunit and their organization are not yet characterized, they are not essential for filament formation because some flagellin defects are compensated by others [26]. The flagellar filament of *Sinorhizobium meliloti* consists of four flagellins, FlaA, FlaB, FlaC, and FlaD, and that of *Rhizobium lupini* contains just three of them FlaA, FlaB, and FlaD. For the flagella of these soil bacteria, FlaA is the principal component, and others are secondary ones [27]. The flagellar filament of *Rhizobium leguminosarum* comprises three major proteins, FlaA, FlaB, and FlaC, and four minor proteins, FlaD, FaE, FlaH, and FlaG [28]. *Agrobacterium tumefaciens* also possesses four flagellins, FlaA, FlaB, FlaC, and FlaD; FlaA and FlaB are abundant in the filament in comparison with FlaC and FlaD, and the swimming ability of *A. tumefaciens* is considerably decreased by a loss of FlaA but not by that of FlaB [29]. *Bradyrhizobium diazoefficiens* has two flagella systems: One is subpolar flagella, of which filament is composed of four flagellins, FliC1, FliC2, FliC3, and FliC4, whereas the other is lateral flagella, of which filament is made up of two flagellins, LafA1 and LafA2 [30]. The bi-polar flagellar filaments of *Campylobacter jejuni* comprise two distinct FlaA and FlaB subunits, both of which share 92.3% sequence identity. The FlaB filament grows first and then FlaA filament grows on the FlaB filament [31]. Consistently, two different flagellins, FlaA and FlaB (86% sequence identity) form the single polar flagellar filament in *Shewanella putrefaciens*, and FlaA forms a proximal part of the filament whereas FlaB makes the remaining portion [32]. The spatial assembly by these two distinct flagellin subunits benefits motility under a various range of environmental conditions [32]. Because the assembly of the flagellar filament by multiple flagellins affects its mechanistic properties for flagellar function in different environments [26,32,33,34,35], the composition of the flagellar filament structure would be optimized for environmental conditions, in which the bacteria live and survive.

### 2.2. Hook and Rod

The *Salmonella* hook is formed by about 120 copies of the hook protein FlgE. *Salmonella* FlgE consists of three domains D0, D1, and D2, arranged from the inner to outer parts of the hook structure and the Dc region connecting domains D0 and D1 [36,37]. The hook forms several supercoils, and axial interactions between a triangular loop of domain D1 and domain D2 are responsible for hook supercoiling [36,38,39]. However, a truncation of neither the triangular loop nor the D2 domain affects the bending flexibility of the hook structure [38]. Since there are gaps not only between D1 domains but also between D0 domains, these gaps make the hook flexible for bending. The amino-acid sequence of FlgE of *C. jejuni* (864 a.a for strain NCTC 11168) is much longer than that of *Salmonella* (402 a.a), and so FlgE of *C. jejuni* has two additional outer domains, D3 and D4, and these two domains are involved in the interaction within and between protofilaments, conferring stiffness and robustness on the *C. jejuni* hook structure to act as a universal joint under highly viscous condition [40].

The bending flexibility of the hook structure is required for the formation of a bundle structure behind the cell body of *E. coli* and *Salmonella* [41,42]. The hook length is also important for maximum stability of the flagellar bundle. Shorter hooks are too stiff to function as a universal joint whereas longer hooks buckle and create instability in the flagellar bundle [43]. The hook length is controlled by the molecular ruler protein FliK, which is secreted via a type III protein export apparatus during hook assembly [44].

The elasticity of the hook is also important for changing swimming direction in *V. alginolyticus*, which is a monotrichous bacterium. When *V. alginolyticus* cell changes swimming from forward to backward by the switching of direction of flagellar motor rotation from CCW to CW, the hook undergoes compression and buckles, resulting in an axis mismatch between the flagellar filament and the cell body to induce a flicking motion of the cell body. As a result, the swimming direction changes by ~90° [45].

The rod is composed of three proximal rod proteins, FlgB, FlgC, FlgF, and the distal rod protein FlgG [46,47]. FliE is postulated to connect the MS ring and the most proximal part of the rod formed by FlgB [48]. These four rod proteins and FliE are well conserved among bacterial species [9,10]. Domains D0 and D1 of *Salmonella* FlgG show high sequence and structural similarities to those of FlgE, thereby allowing direct connection of the rigid rod with the flexible hook [49]. However, one major structural difference between the rod and hook is the orientation of their D1 domains relative to the tubular axis, and so axial packing interactions between domains D1 of FlgG are tight whereas those of FlgE are loose. As a result, such a structural difference is likely to be responsible for the bending rigidity of the rod and flexibility of the hook [49]. The Dc region of FlgG has a FlgG specific sequence (GSS; YQTIRQPGAQSSEQTTLP). Since the GSS insertion into the Dc region of FlgE makes the hook straight and rigid, the GSS contributes to the rigidity on the rod structure [42]. However, since FlgE of *B. subtilis* and *C. jejuni* has the GSS-like sequence in their Dc region [32,34], it remains unknown how the hook of *B. subtilis* and *C. jejuni* can form a curved structure with bending flexibility.

## 3. Type III Protein Export Apparatus

The assembly of the axial structure begins with the rod, followed by the hook and finally the filament. A type III protein export apparatus transports axial component proteins from the cytoplasm to the distal end of the growing flagellar structure to construct the axial structure beyond the cellular membranes [50]. The type III protein export apparatus consists of an export gate complex made of five transmembrane proteins, FlhA, FlhB, FliP, FliQ and FliR, and a cytoplasmic ATPase ring complex consisting of FliH, FliI and FliJ [51,52,53]. The transmembrane export gate complex is located within the basal body MS ring and acts as a H^+^–protein antiporter to couple an inward-directed H^+^ translocation through the export gate with an outward-directed protein export [54,55]. FliP, FliQ, and FliR form a right-handed helical assembly with a 5 FliP to 4 FliQ to 1 FliR stoichiometry inside the MS ring, and FliO is required for efficient assembly of the FliPQR complex [52,56,57]. The FliPQR complex has a central channel with a diameter of 1.5 nm [57]. Since FliP and FliR are likely to interact with FliE [51,57,58], the central channel of the FliPQR complex is postulated to be a protein translocation pathway. FlhA and FlhB associate with the FliPQR complex [52]. FlhA forms a nonameric ring structure through its C-terminal cytoplasmic domain [59,60,61] and forms an ion channel to conduct both H^+^ and Na^+^ [62]. FliH, FliI and FliJ form the cytoplasmic ATPase ring complex with a 12 FliH to 6 FliI to 1 FliJ stoichiometry [63,64,65]. The ATPase ring complex is associated with the basal body through interactions of FliH with FlhA and a C ring protein FliN [66,67,68,69]. The FliI_6_ ring hydrolyzes ATP to activate the transmembrane export gate complex, thereby driving H^+^-coupled flagellar protein export by the export gate [55,70].

## 4. Basal Body Rings

The basal body has multiple ring structures, namely L ring, P ring, MS ring, and C ring [71] (Figure 1c). The L and P rings, which are formed by the lipoprotein FlgH and the periplasmic protein FlgI, respectively, are embedded in the outer membrane and the peptidoglycan (PG) layer, respectively, and they together act as a bearing for the rod. The LP ring complex is missing in the basal body of gram-positive bacteria such as *B. subtilis* [9]. In contrast, the MS and C rings are well conserved among bacterial species [9,10]. The MS ring is composed of the transmembrane protein FliF and is part of a rotor [71]. FliG, FliM, and FliN form the C ring on the cytoplasmic face of the MS ring. The C ring acts not only as a central part of the rotor for torque generation but also as a structural device to switch the direction of motor rotation in *E. coli* and *Salmonella* [71]. Diameters of the LP ring complex, the S ring, the M ring, and the C ring are ~25 nm, ~24.5 nm, ~30 nm, and ~45 nm, respectively, in *Salmonella*.

FliG consists of N-terminal (FliG_N_), middle (FliG_M_), and C-terminal (FliG_C_) domains. FliG_N_ directly associates with the C-terminal cytoplasmic domain of FliF (FliF_C_) with a one-to-one stoichiometry [72]. Inter-molecular interactions between FliG_N_ domains and between FliG_M_ and FliG_C_ are responsible for FliG polymerization on the cytoplasmic face of the MS ring [73,74,75,76]. FliG_C_ is involved in the interaction with the stator protein MotA [77,78,79]. The middle domain of FliM (FliM_M_) binds to FliG_M_ with a one-to-one stoichiometry to form the C ring wall [80]. An EHPQR motif in FliG_M_ and a GGXG motif in FliM_M_ are responsible for the FliG_M_–FliM_M_ interaction. The C-terminal domain of FliM (FliM_C_) shows significant sequence and structural similarities with FliN, and FliM_C_ and FliN together form a doughnut-shaped hetero-tetramer consisting of one copies of FliM_C_ and three copies of FliN, and this hetero-tetrameric block produces a continuous spiral density along the circumference at the bottom edge of the C ring [81]. *B. subtilis* has a *fliY* gene, which shows sequence similarity to both FliM_C_ and FliN, instead of the *fliN* gene [82]. In *B. subtilis*, FliG, FliM and FliY form the C ring in a similar manner to *E. coli* and *Salmonella* C ring structures although the overall structure and dimensions of the *B. subtilis* C ring remain unclear. Interestingly, high-resolution single-molecule fluorescence imaging techniques have revealed rapid exchanges of FliM and FliN labelled with a fluorescent protein between the basal body and the cytoplasmic pool in *E. coli*, suggesting that the C ring is a highly dynamic structure [83,84,85].

The stator units are assembled on the FliG ring (Figure 1c), and so stator–rotor interactions occur about 20 nm away from the center of the C ring in *Salmonella*. The *Salmonella* flagellar motor can accommodate about 10 stator units [86]. A *fliF*–*fliG* deletion fusion significantly shortens the diameter of the C ring, because FliF_C_ and FliG_N_, which together form the inner lobe structure connecting the M and C rings, are missing. It has been shown that the average number of active stator units is two units less in the FliF–FliG deletion fusion motor than in the wild-type motor [87]. This suggests that the diameter of the C ring determines the number of active stator units that can be bound to the motor. This is supported by recent observations that a diameter of the C ring of the *C. jejuni* and *H. pylori* flagellar motors is larger than that of the *Salmonella* C ring, allowing these motors to accommodate more active stator units around the rotor to generate much higher torque [88].

## 5. Stator

### 5.1. Diversity of the Stator Unit

The transmembrane stator unit of the flagellar motor conducts ions and exerts force on the rotor. Based on the coupling ion and sequence similarity, the stator units are classified into three groups: H^+^-coupled MotAB complex, Na^+^-coupled PomAB complex, and Na^+^-coupled MotPS complex [14]. The MotAB complex is composed of four copies of MotA and two copies of MotB and acts as a transmembrane H^+^ channel [89,90]. The PomAB and MotPS complexes form a Na^+^ channel in a way similar to the MotAB complex [91,92,93]. In addition to these stator proteins, bacteria such as *S. meliloti* and *V. alginolyticus* have additional motor proteins. *S. meliloti* possesses three extra motor proteins, namely MotC, MotD, and MotE. MotC stabilizes the periplasmic domain of MotB to facilitate proton translocation through a H^+^ channel of the MotAB complex. MotD binds to FliM for fast rotation, and MotE is involved in folding and stability of MotC [94]. *V. alginolyticus* has MotX and MotY to form the T ring structure located beneath the P ring, and an interaction between PomB and MotX is required for stable localization of PomAB complex around the basal body [9,95].

*V. alginolyticus* and *V. parahaemolyticus* use a single polar flagellum for swimming in low viscous liquid and induce lateral flagella when these *Vibrio* cells encounter solid surfaces [96,97,98]. The polar flagellum utilizes the PomAB complex as a stator unit whereas the lateral flagella use the MotAB complex as a stator unit [99]. *B. subtilis* possesses two distinct H^+^-type MotAB and Na^+^-type MotPS complexes to drive flagellar motor rotation, and these two types of stator units are exchanged in response to changes in external pH, external Na^+^ concentration and viscosity [92,93,100]. Like *B. subtilis*, *Shewanella oneidensis* also utilizes two distinct H^+^-type MotAB and Na^+^-type PomAB complexes in response to changes in the environmental Na^+^ concentration [101].

The MotPS complex of *Bacillus alcalophilus* conducts K^+^ and Rb^+^ in addition to Na^+^ [102]. *Bacillus clausii* has only MotAB complex as a stator unit, and this MotAB complex exhibits the H^+^ channel activity at neutral pH and the Na^+^ channel activity at extremely high pH [103]. The MotAB complex of a spirochete *Leptospira biflexa* has the ability to conduct both H^+^ and Na^+^ in an external pH-dependent manner in a way similar to the MotAB complex of *B. clausii* [104]. These observations suggest that the stator function of these species would be optimized for environmental conditions of their habitats.

### 5.2. Topology of the Stator Complex

**Figure 2:**
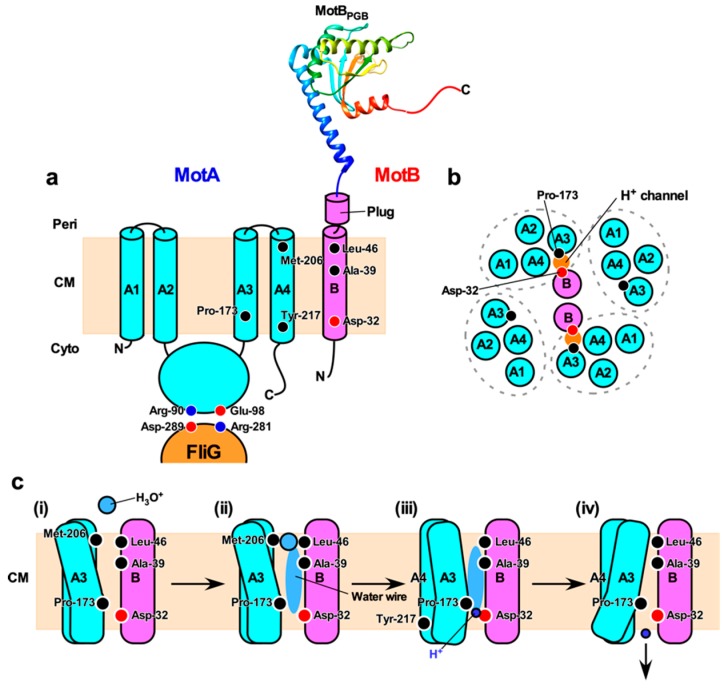
H^+^ translocation mechanism of the flagellar motor. (**a**) Topology of the *E. coli* MotA and MotB and a crystal structure of the peptidoglycan-binding domain of MotB (MotB_PGB_, PDB code: 2ZVY). Highly conserved Arg-90 and Glu-98 residues in the cytoplasmic loop between transmembrane helices 2 (A2) and 3 (A3) interact with conserved Asp-289 and Arg-281 residues of FliG, respectively, to drive motor rotation. Asp-32 of MotB provides a binding site for H^+^. Pro-173, Met-206 and Tyr-217 of MotA and Ala-39 and Leu-46 of MotB are involved in the H^+^ relay mechanism. Cyto, cytoplasm; CM, cytoplasmic membrane; Peri, periplasm. (**b**) Arrangement of transmembrane segments of MotA and MotB. The MotAB complex has two proton channels. Four MotA subunits are positioned with their TM3 (A3) and TM4 (A4) segments adjacent to the MotB dimer, and their TM1 (A1) and TM2 (A2) segments on the outside. (**c**) A plausible model for H^+^ translocation through MotAB stator complex (see text for details).

MotA, PomA and MotP possess four transmembrane helices (TM1, TM2, TM3, and TM4) and a relatively large cytoplasmic loop between TM2 and TM3 and a C-terminal cytoplasmic tail (Figure 2a). MotB, PomB and MotS possess an N-terminal cytoplasmic tail, a single transmembrane helix, and a relatively large C-terminal periplasmic domain containing a conserved peptidoglycan-binding (PGB) motif for anchoring the stator units to the rigid PG layer (Figure 2a). A plausible atomic model of the transmembrane H^+^ channel of the MotAB stator complex derived from *E. coli* has been proposed [105]. The MotAB stator complex has two H^+^ pathways formed by MotA-TM3, MotA-TM4 and MotB-TM (Figure 2b). A highly conserved Asp-32 residue lies near the cytoplasmic end of MotB-TM and plays an important role in the H^+^ relay mechanism [106]. This Asp residue is located on the surface of MotB-TM facing MotA-TM3 and MotA-TM4 [90]. A plug segment in the flexible linker of MotB connecting MotB-TM and the PGB domain binds to the H^+^ channel to suppress massive H^+^ flow through the channel until the MotAB complex associates with the motor. It has been proposed that an interaction between MotA and FliG may induce a detachment of the plug segment form the H^+^ channel to couple the H^+^ flow through the channel to torque generation [107,108].

### 5.3. H^+^ Translocation Mechanism

The maximum rotation rate of the H^+^-driven flagellar motors of *E. coli* and *Salmonella* is reduced with a decrease in the intracellular pH. In contrast, a change in external pH does not affect the maximum motor speed at all. These observations suggest that the intracellular H^+^ concentration affects the rate of the H^+^ flow through the MotAB complex [109,110].

Asp-33 of *Salmonella* MotB, which corresponds to Asp-32 in *E. coli* MotB, is critical for the binding of H^+^ from the cell exterior, and its protonation and deprotonation cycle is directly linked to a torque generation step caused by stator–rotor interactions [111]. The *motB(D33E)* mutation results in a considerable decrease in the rate of H^+^-coupled conformational change of the MotAB complex [112]. Furthermore, the *motB(D33E)* mutation causes not only large speed fluctuations but also frequent pausing of motor rotation at low load. However, neither speed fluctuation nor pausing is seen at high load [112]. These observations suggest that the protonation and deprotonation cycle of Asp-33 of MotB may occur in a load-dependent manner. The dissociation of H^+^ from this Asp-33 residue to the cytoplasm is linked to conformational changes of a cytoplasmic loop of MotA, which is responsible for the interaction with FliG. Molecular dynamics (MD) simulation has predicted that the binding of H^+^ to this Asp residue induces a conformational change of the proton channel to facilitate H^+^ release to the cytoplasm [105]. Two highly conserved residues, Pro-173 of MotA-TM3 and Tyr-217 of MotA-TM4, are involved in such H^+^-coupled conformation changes of the H^+^ channel [113,114,115].

Based on MD simulation of the H^+^ channel of the *E. coli* MotAB complex, the H^+^ translocation through the channel is postulated to be mediated by water molecules aligned along a H^+^ pathway (i.e., water wire). Leu-46 of MotB is assumed to act as a gate for hydronium ion (H_3_O^+^) and then to transfers H^+^ to MotB-Asp32 via the water wire [105]. Mutations at position of Ala-39 of MotB, which resides on the same side as Asp-32 in the H^+^ pathway, impair motility and are partially suppressed by extragenic mutations at Met-206 of MotA [116]. This Met-206 residue is located near the periplasmic end of TM4 and faces the H^+^ pathway [105,117]. The *motA(M206I)* mutation reduces the H^+^ channel activity, thereby reducing motility [118]. Taken all together, the H^+^ translocation mechanism is postulated to be as follows: (i) H^+^ permeates a H^+^ channel in the H_3_O^+^ state through Leu-46 of MotB, (ii) Met-206 of MotA and Ala-39 of MotB are involved in the transfer of H^+^ along the water wire, (iii) H^+^ binds to Asp-32 of MotB, and (iv) the dissociation of H^+^ from Asp-32 of MotB to the cytoplasm is facilitated by a conformational change of the H^+^ channel through Pro-173 and Tyr-217 of MotA (Figure 2c). As a result, the cytoplasmic loop of MotA can interact with FliG to drive flagellar motor rotation [119].

## 6. Torque Generation

### 6.1. Rotation Mechanism

Highly conserved Arg-90 and Glu-98 residues of MotA, which are located in the cytoplasmic loop between TM2 and TM3 of MotA, interact with highly conserved Asp-289 and Arg-281 residues of FliG, respectively (Figure 2a) [77,78,79,120]. These two electrostatic interactions are responsible for efficient stator assembly around the rotor, and the interaction between Glu-98 of MotA and Arg-281 of FliG is likely to be involved in torque generation [79]. H^+^ translocation through the transmembrane H^+^ channel of the MotAB complex allows the cytoplasmic loop of MotA to associate with and dissociate from FliG to drive flagellar motor rotation [119]. However, the energy coupling mechanism of the flagellar motor remains unknown.

### 6.2. Torque-Speed Relationship

**Figure 3:**
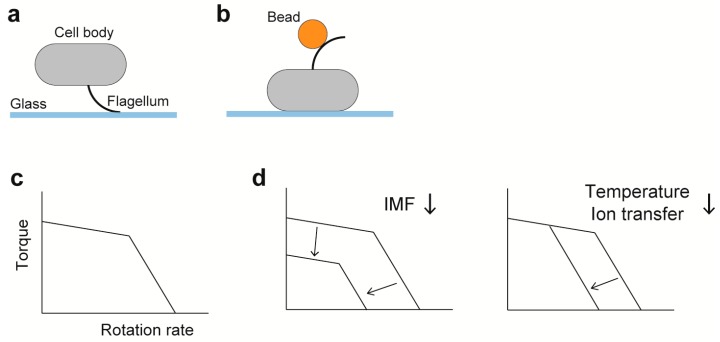
Characterization of the rotation of the flagellar motor. (**a**) Tethered cell assay. (**b**) Bead assay; gold nanoparticles (60–100 nm in diameter) and polystyrene beads (0.2–2.0 μm in diameter) are used. (**c**) A schematic of the torque-speed curve. (**d**) Effects of factors relevant to motor dynamics on the torque-speed curve. Dependence of the curve on the number of stator units is described in the section of *Duty ratio*.

Precise measurements of motor rotation are important to elucidate the torque-generation mechanism of the flagellar motor. Direct evidence that the bacterial flagellum is a rotary motor is obtained by tethered cell assay (Figure 3a), in which the cell body rotates by tethering the filament to a glass surface [121]. The tethered cell assay is a simple method to measure the rotation of the flagellar motor to give fundamental knowledges on the motor mechanism. However, the maximum speed of tethered cells is limited below 20 Hz, because a cell body (~2 μm in length) is extremely large load against the flagellar motor (~45 nm in diameter). To measure the rotational speeds of the *E. coli* flagellar motor over a wide range of external load, bead assay was developed by the Howard Berg laboratory (Figure 3b) [11,122,123]. A bead is attached to a partially sheared sticky flagellar filament lacking domain D3 of flagellin as a probe, and then the rotation of the bead is recoded by a quadrant photodiode or a high-speed camera with high temporal and special resolutions. Therefore, bead assays enable us to investigate output properties of the flagellar motor over the wide range of external load by changing the bead size and medium viscosity. Viscous drags on a bead (*γ*_b_) and a truncated filament (*γ*_f_) are obtained from the bead diameter and the flagellar morphology (filament length and thickness, and helical pitch and radius), respectively, based on a hydrodynamic theory [124,125], and so motor torque (*M*) can be estimated by *M* = (*γ*_b_ + *γ*_f_) · 2π*f*, where *f* is the rotation rate.

Figure 3c shows a schematic diagram of the torque versus speed relationship of the flagellar motor, namely torque-speed curve. The torque-speed curve of the flagellar motor consists of two regimes: a high-load, low-speed regime and a low-load, high-speed regime [11]. As external load is decreased, torque decreases gradually up to a certain speed and then falls rapidly to zero. The rotation rate of the flagellar motor is proportional to IMF over a wide range of external load (Figure 3d) [126,127]. Both deuterium oxide and temperature affect the rotation rate of the *E. coli* motor operating in the low-load, high-speed regime but not in the high-load, low-speed regime (Figure 3d), suggesting that a steep decline of torque seen in the low-load, high-speed regime is limited by the rate of H^+^-coupled conformational changes of the MotAB complex [11,123]. Torque at high load is dependent on the number of active stator units in the motor, whereas the maximum motor speed near zero load is independent of the stator number [122,128,129]. However, recent two biophysical analyses have revealed that the maximum speed near zero load increases with an increase in the number of active stator units in the motor [130,131], suggesting that both torque and speed would be proportional to not only IMF but also to the stator number over a wide range of external load.

### 6.3. Stepwise Rotation

Discretely stepwise movements have been observed in many molecular motors. For example, kinesin, which is an ATP-driven linear motor, moves along a microtubule with steps of 8 nm interval [132]; myosin V on an actin filament shows stepwise movements with 36 nm intervals with 90° random rotation either CCW or CW [133]; and F_1_-ATPase, which is the ATP-driven rotary motor, shows a 120° step, which is further divided into 80° and 40° substeps [134]. Such stepwise movements reflect the elementary process of mechanochemical energy coupling, e.g., 80° and 40° substeps in F_1_-ATPase are coupled with ATP binding and Pi release, respectively, and thus kinetics and dynamics of the step events are important for understanding the motor mechanism. When the flagellar motor labelled with a small bead (diameter: ~100 nm) contains only a single stator unit around a rotor and spins at a few Hz, stepping motions of the motor has been observed. The flagellar motor containing a single stator unit rotates with 26 steps per revolution in both CCW and CW directions [135,136]. Since the number of steps per revolution is consistent with the rotational symmetry of the FliG ring, it is suggested that torque is generated through cyclic association–dissociation of MotA with every FliG subunit along the circumference of the rotor and that such an elementary process is symmetric in CCW and CW rotation. However, it remains unknown how the protonation–deprotonation cycle of Asp-32 of MotB is linked to the cyclic association–dissociation of MotA with FliG.

### 6.4. Duty Ratio

The duty ratio is defined as a fraction of time that a stator unit is bound to a rotor in the mechanochemical cycle of the flagellar motor. The duty ratio is one of the fundamental properties of molecular motors and is an important parameter for understanding the operation mechanism. The duty ratio of the flagellar motor has been discussed based on the dependency of the rotation rate on the number of active stator units in the motor [122,128,129,130,131]. At high load where torque generation against load is a rate limiting step, the rotation rate is proportional to the number of active stator units in the motor regardless of the value of the duty ratio: If the duty ratio is large (~1), the rotation rate is proportional to the sum of the applied torque because multiple stator units work together at the same time; If the duty ratio is small (<< 1), each stator unit works independently and so the probability of torque generation by the motor per a certain period of time is increased with an increase in the number of active stator units in the motor. As a result, the rotational speed of the flagellar motor is proportional to the number of active stator units in the motor. At low load where kinetic processes (e.g., proton translocation and conformational change) are rate limiting steps, the relationship between the rotation rate and the number of active stator units would depend on the duty ratio: If the duty ratio is close to 1, total torque does not affect the rotation rate, and so the rotation rate of the motor does not depend on the number of active stator units in the motor; if the duty ratio is small, the probability of torque generation by stator-rotor interactions is increased with an increment in the stator number. Ryu et al. have shown that the stator number dependence of the rotational speed of the *E. coli* flagellar motor becomes smaller when external load becomes lower [122]. Furthermore, Yuan and Berg have shown that the maximum speed of the *E. coli* motor is independent of the number of active stator units in the motor [128]. Recently, Wang et al. have reported that the maximum speed of the *E. coli* motor near zero load is constant although the number of active stator units varies [129]. These three studies have suggested that the duty ratio of the flagellar motor seems to be large. Assuming that the flagellar motor has a high duty ratio, theoretical studies can reproduce the output properties of the flagellar motor such as a torque-speed curve [137,138,139,140,141]. In contrast, a recent study using a hybrid motor containing both H^+^-type and Na^+^-type stator units in *E. coli* cells has shown that the maximum speed of the hybrid flagellar motor near zero load varies with the number of active stator units in the motor [130]. This observation is supported by recent observation that the zero-torque speed of the *Salmonella* flagellar motor depends on the number of active stator units in the motor [131]. These suggest that the duty ratio of the flagellar motor operating at low load is smaller than the previous thought. By removing the high duty ratio constraint from the theoretical model, it is also possible to reproduce the stator-number-dependent rotational speed close to zero load. This physical model also predicts that the duty ratio will become larger with increase in the number of active stator units when the motor operates at low load and that a high duty ratio will be required for the motor to processivity generate much larger torque at high load [142]. Thus, the duty ratio of the flagellar motor is currently controversial, and hence further experimental verification over a wide range of external load will be necessary.

## 7. Switching of Direction of Flagellar Motor Rotation

### 7.1. Conformational Changes for Reversal of Motor Rotation

*E. coli* and *Salmonella* cells sense temporal changes in chemical concentrations of attractants and repellents via transmembrane chemoreceptors (methyl-accepting chemotaxis proteins, MCP) localized near the cell pole [143]. The binding of repellent to MCP induces auto-phosphorylation of CheA via the adopter protein CheW, and then CheA-P transfers a phosphate to the response regulator CheY. The binding of the phosphorylated form of CheY (CheY-P) to FliM and FliN induces the structural remodeling of the C ring responsible for the switching of direction of flagellar motor rotation from CCW to CW. The relationship between the switching frequency and CheY-P concentration shows a sigmoid curve with a Hill coefficient of ~10 [144]. This switching Hill coefficient value is larger than the Hill coefficient estimated from the binding affinity of CheY-P for the motor [145,146]. This suggests that CheY-P-dependent structural remodeling of the C ring occurs in a highly cooperative manner.

**Figure 4:**
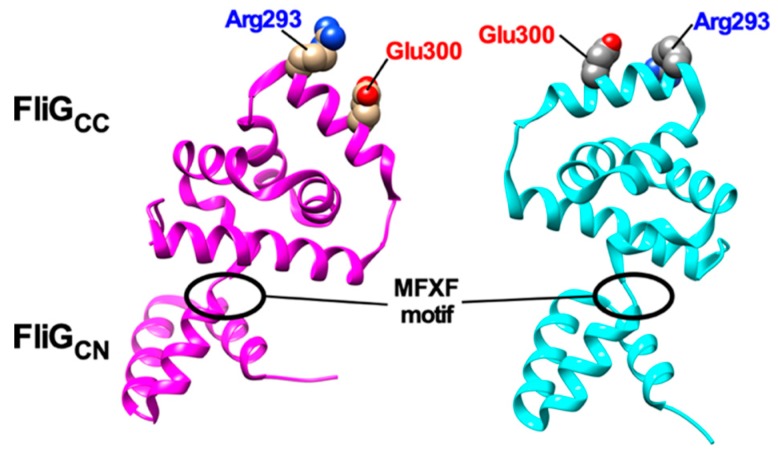
Structural comparisons between 3USY (cyan) and 3USW (magenta) structures of *Helicobacter pylori* FliG. Conformational rearrangements of the conserved MFXF motif induces a 180° rotation of FliG_CC_ relative to FliG_CN_ to reorient Arg-293 and Glu-300 residues, which correspond to Arg-281 and Asp-289 of *E. coli* FliG, respectively.

Since the elementary process of torque generation by stator-rotor interactions is symmetric in CCW and CW rotation, FliG_C_, which contains highly conserved Arg-281 and Asp-289 residues involved in the interaction with MotA, is postulated to rotate 180° relative to MotA [136]. FliG_C_ has a highly flexible MFXF motif between FliG_CN_ and FliG_CC_ subdomains and so the MFXF motif allows FliG_CC_ to rotate 180° relative to FliG_CN_ to reorient Arg-281 and Asp-289 residues in FliG_CC_ to achieve the symmetric elementary process of torque generation in both CCW and CW rotations (Figure 4) [147,148].

Helix_MC_ is a helical linker connecting FliG_M_ and FliG_N_ and plays an important role in directional switching of the flagellar motor [149]. A deletion of three residues in the N-terminal end of Helix_MC_ (Pro-Ala-Ala, PAA) locks the flagellar motor in the CW state even in the absence of CheY-P [149]. The PAA deletion causes conformational rearrangements of the FliG_M_–FliM_M_ interface to induce a detachment of Helix_MC_ from the interface. Furthermore, this PAA deletion induces a 90° rotation of FliG_CC_ relative to FliG_CN_ through the MFXF motif in solution [75,76,149]. This is supported by in vivo site-directed crosslinking experiments [150]. Recent cryoEM image analyses have shown that inter-subunit spacing between C ring proteins are closer in the C ring of the CW motor than in that of the CCW motor [87], suggesting that the binding of CheY-P to FliM and FliN significantly affects inter-molecular interactions between the C ring proteins. Therefore, it is possible that the binding of CheY-P to FliM and FliN changes inter-molecular FliM_M_–FliM_M_, FliM_C_–FliN and FliG_M_–FliM_M_ interactions in the C ring to induces the dissociation of Helix_MC_ from the FliG_M_–FliM_M_ interface, thereby affecting inter-molecular FliG_M_–FliG_CN_ interactions to allow FliG_CC_ to rotate 180° relative to FliG_CN_ through a conformational change of the MFXF motif (Figure 4).

In *E. coli* and *Salmonella*, the binding of repellent to MCP elevates the cytoplasmic CheY-P level, thereby increasing the probability that the motor spins in CW direction. In contrast, the chemotaxis signaling pathway and response are known to diverse among bacterial species. In *B. subtilis*, CheY-P acts in the opposite way to induce CCW rotation. The binding of attractant to MCP of *B. subtilis* facilitates phosphorylation of CheY, and CheY-P binds to FliM to switch motor rotation from CW to CCW [151]. *Rhodobacter sphaeroides* possesses six CheY proteins, CheY_1_ to CheY_6_. The decreased attractant concentration increases the cytoplasmic CheY_3_-P, CheY_4_-P, and CheY_6_-P levels, and the binding of CheY_6_-P to FliM stops motor rotation with the support of CheY_3_-P and CheY_4_-P [152].

### 7.2. Conformational Spread for Cooperative Switching

**Figure 5:**
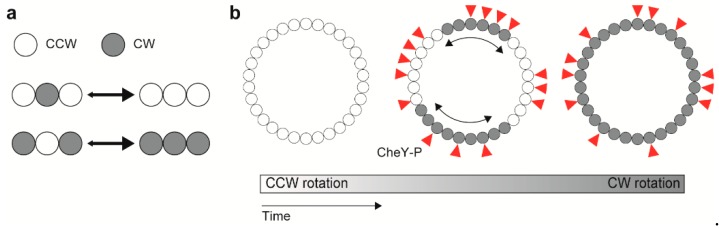
Model for cooperative switching between counterclockwise (CCW) and clockwise (CW) rotations. (**a**) Interaction between adjacent rotor subunits. (**b**) Conformational spread upon CheY-P binding.

Cooperative flagellar switching can be reproduced by an Ising-type model assuming allosteric cooperativity of the conformational change in C ring subunits [153]. The model assumes four states for each subunit, determined by whether a subunit conformation is placed in either the CCW or CW state with or without CheY-P bound. Assuming that homogeneous states of adjacent subunits (e.g., CCW-CCW-CCW or CW-CW-CW) are more stable than heterogeneous ones (e.g., CCW-CW-CCW or CW-CCW-CW), the directional switching is mediated by conformational changes in C ring subunits that extend from subunit to subunit via inter-molecular interactions between nearest adjacent subunits (Figure 5) [153]. The model prediction was verified by simultaneous measurements of motor rotation and a turnover of CheY labelled with a green fluorescent protein (GFP) between the motor and the cytoplasmic pool, showing that, in spite of the switch complex contains ~34 FliM subunits, the binding of about 13 CheY-P molecules can reverse the motor [154].

The switching rate increases until the motor speed reaches ~150 Hz, and then decreases with further increase in the rotation rate [155,156]. The conformational spread model also explains a speed (load) dependent switching frequency by assuming the effect of mechanical force on the switching rate, which each stator unit applies force on the FliG subunit in the C ring [140]. The conventional Ising-type conformational spread model, which is an equilibrium model sufficient for detailed balance, shows exponentially decayed distributions of the duration time for CCW or CW rotation. Such exponential duration-time distributions have been observed experimentally, suggesting the equilibrium switching system. Recently, Wang et al. have measured the CCW and CW durations at various conditions of load, PMF, and the number of active stators and have shown non-exponential shaped distributions in a torque-dependent manner. The results suggest that the flagellar switch could be a non-equilibrium system rather than an equilibrium system under certain conditions, and that motor torque is a key factor for breaking detailed balance. Furthermore, the directional switching of the flagellar motors working under non-equilibrium conditions (e.g., at high load) can occur at lower CheY-P level compared to those placed under equilibrium conditions, suggesting that the binding affinity of the flagellar motor for CheY-P is enhanced by applied force [157]. Thus, the switching of direction of flagellar motor rotation is controlled not only by the chemotactic signaling pathway but also by the mechanical force [140,157].

## 8. Stator Assembly

**Figure 6:**
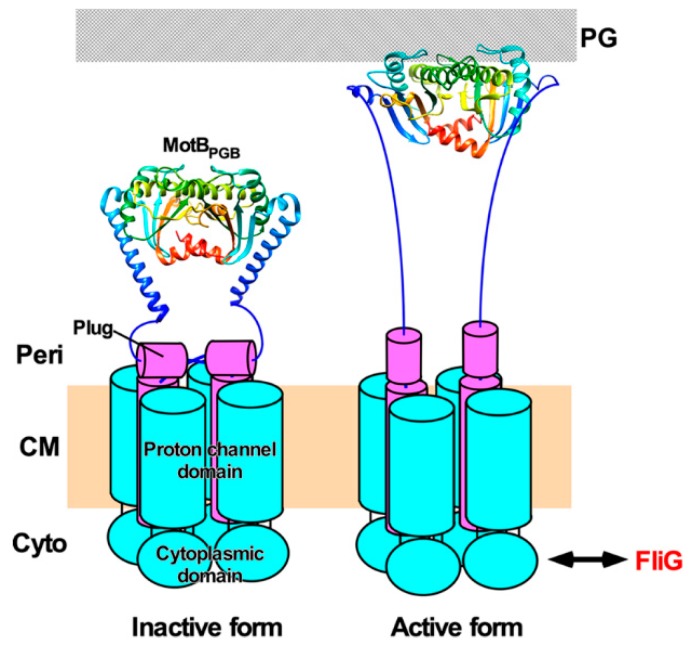
Activation mechanism of the H^+^-type MotAB complex. The MotAB complex consists of at least three structural parts: a cytoplasmic domain, a transmembrane ion channel and a peptidoglycan-binding domain [MotB_PGB_, PDB codes: 2ZVY (left panel) and 5Y40 (right panel). When the MotAB complex adopts a compact conformation, a plug segment of MotB binds to a transmembrane H^+^ channel to suppress massive H^+^ flow (left). When the MotAB complex encounters a rotor, electrostatic interactions between the cytoplasmic domain of MotA and FliG trigger the dissociation of the plug segment from the channel, followed by partial unfolding of the N-terminal portion of MotB_PGB_ to allow MotB_PGB_ to bind to the peptidoglycan (PG) layer. As a result, the MotAB complex becomes an active H^+^-type stator unit to drive flagellar motor rotation (right).

The PGB domains of MotB (MotB_PGB_) and PomB (PomB_PGB_) bind to the PG layer to allow the MotAB and PomAB complexes to become an active stator unit around a rotor [93,158,159]. The N-terminal portions of MotB_PGB_ and PomB_PGB_ adopt a compact conformation in their crystal structure, but are structurally flexible to allow them to adopt an extended conformation as well (Figure 6). Structure-based mutational analyses of MotB_PGB_ and PomB_PGB_ have suggested that a 5 nm extension of the PGB domain from the transmembrane ion channel is required for the binding of MotB_PGB_ and PomB_PGB_ to the PG layer (Figure 6) [158,160]. Recently, such a 5 nm extension process of the PGB domain of MotS (MotS_PGB_) of *B. subtilis* has been directly visualized by high-speed atomic force microscopy [93]. The 5 nm extension of MotS_PGB_ is divided into at least two steps [93]. The first 2.5 nm extension step is caused by a detachment of a flexible linker connecting MotS_PGB_ with MotS-TM from the transmembrane Na^+^ channel of the MotPS complex, and the second 2.5 nm extension step results from an order-to-disorder transition of the N-terminal portion of MotS_PGB_. Consistently, the *motB(L119P)* mutation in MotB_PGB_ induces an extended conformation of the N-terminal portion of MotB_PGB_ [159]. Interestingly, the *motB(L119P)* mutation increases not only the PGB binding activity of MotB_PGB_ [159] but also the proton channel activity of the MotAB complex [107]. Therefore, it seems likely that proper positioning of an inactive MotAB complex around the rotor via stator–rotor interactions triggers a detachment of the flexible linker from the H^+^ channel, followed by a structural transition of the N-terminal portion of MotB_PGB_ from the compact to extended forms to become an active stator unit in the motor [159] (Figure 6).

The flagellar motor can accommodate about 10 stator units around a rotor in *E. coli* and *Salmonella* when the motor operates at high load [161]. High-resolution single molecule imaging techniques have revealed exchanges of the MotAB complex labelled with GFP between the basal body and the membrane pool during rotation at a rate constant of 0.04 s^−1^, indicating that the dual time of a given stator unit is about 0.5 min. This suggests that the interaction of MotB_PGB_ with the PG layer is highly dynamic, thereby allowing the MotAB complex to alternate in attachment to and detachment from the motor during motor rotation [162]. Interestingly, when external load becomes low enough, only a few stator units work around the rotor to drive motor rotation [163,164,165]. This suggests that such a dynamic assembly–disassembly process of the stator complex occurs in a load-dependent manner.

The number of active stator units can be estimated by resurrection experiments, in which time traces of the rotational speed of a single flagellar motor usually show stepwise speed increments and decrements. Each increment reflects the incorporation of a single MotAB complex around the rotor to become an active stator unit in the motor whereas each decrement unit reflects the disassembly of the MotAB stator complex from the rotor [163,164,165]. A deletion of a flexible linker connecting MotB-TM and MotB_PGB_ results in a rapid decrease in the number of active stator units in the motor compared to the wild-type motor, suggesting that this flexible linker of MotB modulates the binding affinity of MotB_PGB_ for the PG layer in a load-dependent manner [166]. Certain mutations in the cytoplasmic loop of MotA, which interacts with FliG, significantly affect the mechano-sensitivity of the MotAB complex, thereby causing distinct load-dependent assembly and disassembly dynamics compared to the wild-type. This suggests that the cytoplasmic loop of MotA may sense a change in external load through the interaction with FliG to control the number of active stator units around the rotor [86].

How does the cytoplasmic loop of MotA transmit the mechanical signal to MotB_PGB_ associated with the PG layer to coordinate the number of active stator units in the motor in response to changes in external load? Nord et al. have reported that the dissociation rate of the MotAB stator complex becomes slower with an increase in applied force, thereby increasing the bound lifetime of each active stator unit incorporated into the motor, and that an abrupt relief from the stall makes the dissociation rate much faster, thereby decreasing the bound lifetime [130]. As a result, the average number of active stator units in the motor is maintained about 10 in the high-load, low speed regime whereas the stator number is decreased from 10 to a few when external load becomes quite low. Recently, it has been shown that a turnover process of the stator unit is divided into two distinct, slow (the rate constant of ~0.008 s^−1^) and fast (~0.2 s^−1^) steps [167]. Although the slow step called “hidden state” is not yet clarified, the fast step is assumed to reflect a rapid conformational change of MotB_PGB_ to become an active stator unit.

The assembly and disassembly dynamics of the stator complex are also affected by changes in the extracellular ion concentration [93,101,118,168,169]. The Na^+^-coupled PomAB and MotPS complexes can be assembled into a motor when the external Na^+^ concentration is high enough [93,101,168]. How do the PomAB and MotPS complex sense external Na^+^? High-speed atomic force microscopy with high special and temporal resolutions has revealed that MotS_PGB_ adopts a folded conformation in the presence of 150 mM NaCl, but becomes denatured when the external Na^+^ concentration is less than 150 mM NaCl. These direct observations suggest that MotS_PGB_ functions as a Na^+^ sensor to efficiently promote the assembly and disassembly of the MotPS complex with the motor in response to changes in external Na^+^ concentration [93].

## 9. Conclusions and Perspectives

The flagellum of *E. coli* and *Salmonella* is a supramolecular rotary motor powered by an inward-directed H^+^ translocation through a transmembrane H^+^ channel of the MotAB stator complex and can spin in both CCW and CW directions without changing the direction of H^+^ flow. The flagellar motor is conserved among bacterial species, but the flagellar structure has adopted to function in various environments of the habitant of bacteria [9,10]. The structure of the rod, hook and filament and their mechanical properties are understood at near atomic resolution. Because structural information on the rotor and stator is still limited, it remains unknown how the transmembrane stator complex conducts ions and exerts force on the rotor, how the rotor switches between the CCW and CW states in a highly cooperative manner, and how the stator complex senses external ion concentration to become an active stator unit around the rotor. To clarify these remaining questions, high-resolution structural analyses of the rotor and stator would be required.

The elementary process of the flagellar motor is visualized to be composed of a step and a dwell [135,136]. Since the dissociation rate of the stator unit becomes much faster at low load than at high load, the number of active stator units in the motor is decreased from 10 to a few when external loads become low enough [130]. Although the duty ratio of the flagellar motor seems to be small, the flagellar motor containing only a few stator units can processively generate torque for high-speed rotation near zero load. However, it remains unknown how the H^+^ translocation process is linked to a torque generation step by stator-rotor interactions and how cyclic association–dissociation of MotA with every FliG subunit along the circumference of the rotor allow the motor to spin at about 300 revolutions per second in a highly processive manner. Much more precise measurements of the rotational speed of the flagellar motor near zero load would be essential to advance our mechanistic understanding of the energy coupling mechanism of the flagellar motor.
